# Practical application of ontologies to annotate and analyse large scale raw mouse phenotype data

**DOI:** 10.1186/1471-2105-10-S5-S2

**Published:** 2009-05-06

**Authors:** Tim Beck, Hugh Morgan, Andrew Blake, Sara Wells, John M Hancock, Ann-Marie Mallon

**Affiliations:** 1 MRC Harwell, Harwell Science and Innovation Campus, Oxfordshire, OX11 0RD, UK

## Abstract

**Background:**

Large-scale international projects are underway to generate collections of knockout mouse mutants and subsequently to perform high throughput phenotype assessments, raising new challenges for computational researchers due to the complexity and scale of the phenotype data. Phenotypes can be described using ontologies in two differing methodologies. Traditionally an individual phenotypic character has either been defined using a single compound term, originating from a species-specific dedicated phenotype ontology, or alternatively by a combinatorial annotation, using concepts from a range of disparate ontologies, to define a phenotypic character as an entity with an associated quality (EQ). Both methods have their merits, which include the dedicated approach allowing use of community standard terminology, and the combinatorial approach facilitating cross-species phenotypic statement comparisons. Previously databases have favoured one approach over another. The EUMODIC project will generate large amounts of mouse phenotype data, generated as a result of the execution of a set of Standard Operating Procedures (SOPs) and will implement both ontological approaches to capture the phenotype data generated.

**Results:**

For all SOPs a four-tier annotation is made: a high-level description of the SOP, to broadly define the type of data generated by the SOP; individual parameter annotation using the EQ model; annotation of the qualitative data generated for each mouse; and the annotation of mutant lines after statistical analysis. The qualitative assessments of phenodeviance are made at the point of data entry, using child PATO qualities to the parameter quality. To facilitate data querying by scientists more familiar with single compound terms to describe phenotypes, the mappings between the Mammalian Phenotype (MP) ontology and the EQ PATO model are exploited to allow querying via MP terms.

**Conclusion:**

Well-annotated and comparable phenotype databases can be achieved through the use of ontologically derived comparable phenotypic statements and have been implemented here by means of OBO compatible EQ annotations. The implementation we describe also sees scientists working seamlessly with ontologies through the assessment of qualitative phenotypes in terms of PATO qualities and the ability to query the database using community-accepted compound MP terms. This work represents the first time the combinatorial and single-dedicated approaches have both been implemented to annotate a phenotypic dataset.

## Background

The laboratory mouse is widely considered by scientists in many domains as the primary model organism for the investigation of human disease. Over the years research using mouse models has aided the understanding of fundamental biological processes as well as playing a major role in identifying genetic loci involved with disease susceptibility [1]. A key component to the investigation of mouse models is the characterisation of their phenotype and the relationship to the underlying genotype.

The classification and analysis of mouse phenotypes has been performed in laboratories for many years, in essence since the beginning of mouse genetics. The majority of this characterisation would have been on a small number of mouse models associated with a particular phenotype or disease model. These mutants would have largely been from spontaneous mutations arising in mouse laboratories across the world. The number of mutants that can be generated is greatly increased by using the alkylating agent N-ethyl N-nitrosourea (ENU), considered one of the most potent mutagens in mice, which is used to induce point mutations randomly across the genome. A key switch from the analysis of a handful of mutants in individual laboratories or specialised consortiums to the analysis of a large collection of mutants was therefore the initiation of ENU mutagenesis programmes [2].

More recently new more elaborate techniques such as gene targeting and conditional gene trapping have facilitated gene driven mutagenesis programmes which aim to analyse the functions of specific genes and ultimately of every gene in the mouse genome. Four projects (EUCOMM [3], funded by the European Commission; KOMP , funded by the National Institutes of Health; NorCOMM , funded by GenomeCanada and the TIGM initiative at the Texas A&M Institute for Genomic Medicine ) are currently underway to produce these large collections of mouse mutants [4].

The EUMODIC project (), which aims to provide a phenotype assessment of up to 650 of these knockout lines, was set up as a pilot project for the large-scale phenotypic assessment of these mutant resources. The scale and complexity of the data that will be generated by these projects, together with their analysis and interpretation, raises new challenges for research in computation. The two primary computational challenges arising are the standardised description of the phenotyping procedure and the rigorous use of ontologies to describe the data in ways which enable the data to be accessible to scientists as well as computationally interpretable.

To date, two differing approaches have been adopted to annotate mouse phenotype data with bio-ontologies. Either a single dedicated ontology of compound terms can be employed or an annotation can be built using terms from a number of distinct ontologies to form a more complex expression to describe an aspect of an organism's phenotype [5]. The Mammalian Phenotype (MP) ontology [6] is an example of a single dedicated phenotype ontology and the PATO model [7] of defining phenotypes in terms of an entity (E) which has a quality (Q) to build EQ annotations is an example of the combinatorial approach.

PATO originated as a species neutral ontology of attributes and values and works in conjunction with specialised domain ontologies that define the entity under observation, which can be, for example, an anatomical structure, a biochemical molecule or a biological process. Originally, a tripartite structure consisting of an entity + attribute + value were used to describe an individual phenotypic character of an organism, for example 'eye' + 'color' + 'red'. However, PATO has progressed to an ontology of phenotypic qualities [8], distinguishing between qualities which inhere in physical objects and qualities which inhere in processes. The two hierarchies of attributes and values have been reconstructed into a single hierarchy of qualities, so forming bipartite phenotypic descriptions of 'entity' + 'quality'. So, in keeping with the previous example, the concept 'red' is now related to 'color' within PATO, using the relationship type *is_a*. Therefore, an identical observation to that in the previous example would be annotated using the EQ structure as 'eye' + 'red'.

The Open Biomedical Ontologies (OBO) family of ontologies allow for the categorisation of individual terms into slim families. Slims are cut-down versions of the whole ontology, containing only a subset of the terms. In the case of the Gene Ontology (GO) they allow for a broad overview of the ontology content without the detail of the finer-grained terms [9], however they also allow for terms at all levels of specificity within an ontology to be grouped if they belong to a more specific type. The ability to assign a PATO concept as an attribute or value has been retained through the ability to assign a concept membership of the PATO attribute slim or value slim categories. The maintenance of the attribute and value distinction has facilitated our application of PATO. PATO's species neutrality lends itself to the formation of comparable cross-species and cross-database EQ phenotypic statements. A mouse kinked tail phenotype can be used as an example to illustrate how MP and the PATO model can each be used to assign an annotation. MP defines this phenotype using the single term "kinked tail" (MP:0000585) and PATO is used to assign a quality to the mouse anatomical entity defined by the Mouse adult gross Anatomy (MA) ontology [10] to form the annotation E: tail (MA:0000008) and Q: kinked (PATO:0001798).

MP has been widely implemented within database resources with the Mouse Genome Database [11] and the Rat Genome Database [12] providing associations between genes and MP terms. However, although recently used for the description of phenotypes observed during screens of the tropical freshwater fish, zebrafish [13], there has, up to now, not been any such comprehensive implementation of the PATO combinatorial approach within any mammalian phenotype related informatics resources.

The phenotypic assessment used in EUMODIC consists of a selection of 22 Standard Operating Procedures (SOPs), from the European Mouse Phenotyping Resource of Standardised Screens (EMPReSS, ) [14,15] organised into two primary phenotyping pipelines, which consist of a series of SOPs individually timetabled to be implemented over a period of seven weeks. The collection of 22 SOPs is termed EMPReSSslim  and includes a wide range of procedures collecting phenotype data from the mouse biological domains of morphology and metabolism; cardiovascular system morphology and physiology; bone density and morphology; neurobehavioral and sensory development; haematology and clinical chemistry; and allergy and immunity responses. Cohorts of both mutant and baseline control mice pass through the pipelines.

As a result of carrying out an individual SOP, quantitative data (e.g. blood pressure measurement), qualitative data (e.g. coat color description) or a combination of quantitative and qualitative data (e.g. cornea opacity description and the precise opacity level measurement) can be returned. This raw phenotyping data on both mutant and background mouse strains is generated in four mouse clinics across Europe and the data generated for each mouse is captured and stored in the EuroPhenome resource ([16]. As mentioned previously it is vital that the descriptions of the SOPs support the unambiguous interpretation and reuse of the data. To ensure this, all data captured in EuroPhenome conforms to the Minimal Information for Mouse Phenotyping Procedures (MIMPP) standard and is captured using its associated XML data format [17].

Our task is to establish how bio-ontologies can be utilised in this project to ensure that the raw individual mouse qualitative data captured are standardised and consistent across the centres and show how quantitative data can be annotated and analysed with ontologies. Here we present how we are currently implementing these phenotype ontologies in this project, issues we have identified, and how we envisage automatically inferring and annotating mouse phenotypic ontology terms to mutant lines.

## Implementation and results

**Figure 1:**
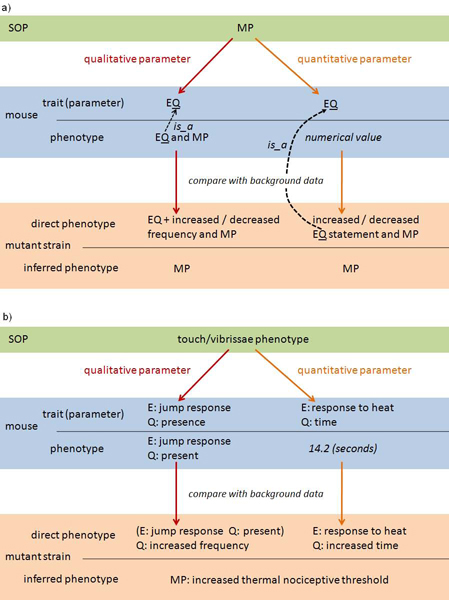
**Levels of ontology annotation of mouse phenotype data**. The relationships between the levels of annotation are shown along with real annotations taken from the Hotplate SOP. a) The SOP is annotated using MP. Each parameter, representing a mouse trait, is defined using EQ. At the point of annotating individual mouse data, qualitative and quantitative parameters are handled differently. Qualitative parameters have a quality assigned to them, with a child-to-parent "is a" relationship to the parameter quality, and may be described using MP where a relevant concept exists. Quantitative parameters have a numerical value assigned to them. After comparison of the mutant line (cohort of individual mice) to the baseline data, statistically significant lines are annotated dynamically. Qualitative EQ data is annotated as being present at an increased or decreased frequency and quantitative data annotation using an increased or decreased based EQ statement, where the quality is a child of the parameter quality. In both cases, if a relevant MP term exists to define the direct phenotype it is assigned. The annotation of inferred phenotypes using MP terms is explained in the Discussion. b) Ontology terms are used to define two Hotplate SOP parameters. Example data is used to illustrate possible annotations of the mouse and the mutant strain. The annotations of the direct phenotypes allow association with an inferred phenotype.

The ontological annotation and definition of mammalian phenotype data was undertaken on four levels: the overall annotation of the SOP's purpose; the annotation of individual quantitative measured parameters; the definition of the qualitative parameters and the allowed results; and the computational annotation of mutant lines with phenotype terms after statistical analysis within EuroPhenome (Figure 1). A distinction is drawn between qualitative and quantitative phenotype data as the annotation of these two classes of data is handled differently.

### Overall annotation of the SOP's purpose

Each EMPReSS SOP has associated with it a list of standardised parameters that are mesasured through the implementation of the SOP. The EMPReSSslim SOPs were annotated using high-level MP terms to give a general description of the purpose of the procedure and provide a global summary of all parameters within the SOP (). This ontological annotation complies with the MIMPP standard. The assumption is therefore that the resulting MP annotation of all measured parameters in a SOP will be children of this high level term. For example, the grip strength SOP is annotated to the MP term "behavior/neurological phenotype" (MP:0005386) with regard to its purpose. The grip strength SOP contains two parameters which involve the measurement of the forelimb grip strength in isolation in grams, and the measurement of forelimb and hindlimb grip strength combined in grams (for further details of this SOP see ). If statistically significant phenotypes were observed for either of these parameters the mutant line in questions would be annotated to the MP term "abnormal grip strength" (MP:0001515), which *is_a *behavior/neurological phenotype within MP.

### Annotation of individual measured parameters

The directly observed phenotypes identified by individual measured parameters are defined using the EQ combinatorial approach in collaboration with scientists with expert knowledge in each domain. Entities are defined from a range of OBO Foundry ontologies, including the Mouse adult gross Anatomy (MA) ontology, Chemical entities of biological interest (ChEBI) [18] and the biological process branch of GO [19]. The PATO qualities used to define the parameter are members of the PATO attribute slim category.

Quantitative parameters have a numerical value assigned to them which are stored in EuroPhenome. After comparison of the mutant line (cohort of individual mice) to the baseline data, statistically significant lines are annotated dynamically. This is illustrated in Figure 1.

An overriding factor in the process of annotating parameter definitions, especially while defining parameters for qualitative data, was making the parameters intuitive to the phenotyping scientists who would be interacting with the local Laboratory Information Management System (LIMS) in mouse clinics. A desirable situation with respect to data accuracy and consistency would be one where original LIMS entries could be imported directly into the EuroPhenome data schema, therefore requiring that non-informaticians should be able to work seamlessly with ontologies. Given the large number of entries into the local LIMS which would be required for a single SOP during the lifetime of EUMODIC and the associated time cost, it was essential that the practical implementation of ontology terms to define parameters was accessible to phenotyping scientists. As a result of this process it was discovered that ontology classes and metadata did not exist to define anatomical entities using terminology that was understandable to phenotyping scientists. These omissions were dealt with by either proposing new terms for the MA ontology, submission of synonyms of existing terms or requests for term definitions.

### Definition of qualitative data

Qualitative data, for example the data generated from the implementation of a dysmorphology SOP, require the objective analysis of data at the point of entry. Qualitative phenotypes, for example variations in coat colours, are compared to wild-type mice and the phenotyper responsible for making the comparison must first make the decision as to whether a mouse is different and if it is, how it is different. The use of ontologies in capturing qualitative data at the point of data entry is desirable, since it reduces the ambiguity associated with interpreting free-text and the subsequent mapping to an ontological structure. For this reason the allowed values that could be assigned to a qualitative parameter were restricted to PATO qualities, specifically qualities that were child terms to the parameter defining quality and members of the PATO value slim category. This process, in unison with the definition of parameters, was carried out in collaboration with phenotyping scientists in EUMODIC.

**Figure 2:**
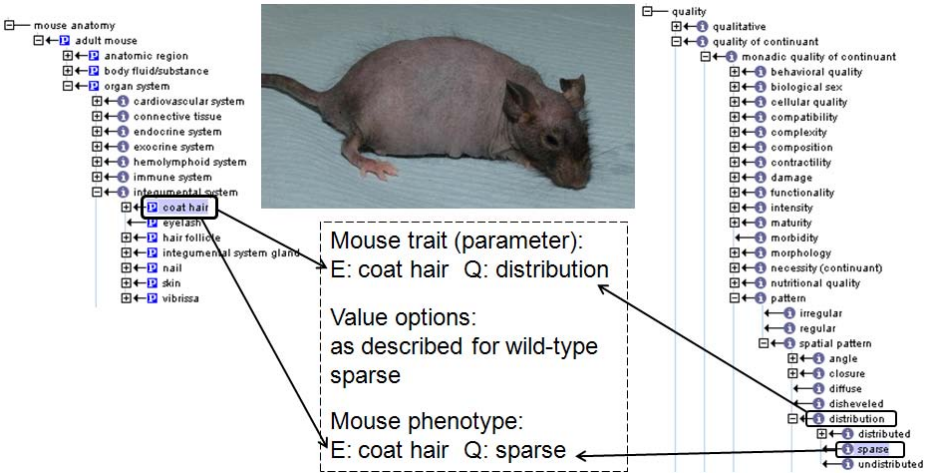
**Relationship between qualitative parameter and data**. An example dysmorphology parameter and the corresponding value options are shown. The central photo shows a mouse with a sparse distribution of coat hair. A portion of the MA ontology is shown on the left and a portion of PATO (Revision 1.118)  is shown on the right (visualised used OBO-Edit [21]). The highlighted terms are used to define the coat hair distribution parameter and the resulting phenotype annotation, illustrating the relationship between the parameter quality and the phenotype quality.

The dysmorphology EMPReSS SOP describes the method to identify morphological abnormalities and will be used here as an example of the ontology based definition of qualitative data. The measured parameters are divided into 8 observable domains to correspond to the defined observable areas within the SOP (whole body, coat hair, skin, head, external genitalia, forelimb, hindlimb, tail). Each observable domain contains related traits to be assessed during the SOP. The traits are defined using MA ontology entities and PATO attribute slim qualities. The list of possible options for each parameter is given, where these are child qualities to the parameter qualities (i.e. PATO value slim qualities) as illustrated in Figure 2. For each background strain a "wild-type fact sheet" contains value qualities for each parameter. When phenotyping mutant mice an assessment of pheno-deviance is made by making a comparison to the background strain mice. Only where a mutant phenotype is identified is it recorded, with all parameters having the value option "as described for wild-type" set as the default value. This is structured so that only a single value is submitted for an individual parameter, so only a single qualitative assessment is made for each parameter. This leads to an extensive list of 181 parameters, which can seem a daunting task to a phenotyping scientist required to enter the data for the many mice phenotyped during the EUMODIC high-throughput screens. However, due to the hierarchical nature of the parameter list, inherited from the Directed Acyclic Graph (DAG) structure of the MA ontology, which define the entities, an expandable and collapsible hierarchical user interface can be implemented in local LIMS, whereby value options can be inherited by parental terms, based on the value submitted for a child term. For example, an observation of increased eye size (E: eye, Q: increased size) when recorded in the LIMS would automatically infer that the parental parameter involving the qualitative assessment of the head would be recorded as "abnormal".

### Computational annotation of inferred phenotypes

As described above, qualitative mouse phenotypes are annotated with ontologies, whereas numerical values are submitted for quantitative mouse parameters, for example the concentration of blood haemoglobin, the time elapsed before the first reaction of a mouse on a hot plate, or the weight of a mouse heart. A cohort of mice (at least seven individuals for the purpose of the EUMODIC screens) each have the same mutation and thus represent a mutant line. Statistical comparisons of mutant lines compared to baseline inbred strains are made for all parameters in EuroPhenome and phenodeviants identified. Categorical qualitative data and quantitative data are subject to specialised statistical tests, with for example, the Chi-squared test used for categorical data and the t-test or the Mann-Whitney U test used for quantitative data. Phenodeviants, which display significantly different values, are then objectively annotated with MP and EQ terms using the parameter annotations described above (Figure 1). The annotation of mutant lines depends on the qualitative or quantitative nature of the parameter. Qualitative parameters are annotated as having the EQ annotation occurring at a higher or lower frequency compared to the baseline stain, thus the EQ annotation is itself annotated as being present at an "increased frequency" (PATO:0000380) or a "decreased frequency" (PATO:0000381). Quantitative data is assigned an EQ annotation after statistical analysis where the quality is a child to the parameter quality and states an increase or decrease compared to the baseline strain. Where equivalent MP concepts exist, these are also used to annotate the direct phenotype. These inferred phenotypes (which may alter over time as new data enter the databases and sample sizes change) can be utilised by users of EuroPhenome to identify mutants of interest and the scientists can then obtain the raw data to determine from their expert knowledge or future analysis that the inferred phenotype is correct.

A distinction is made in the annotation of mutant lines between a direct phenotype and an inferred phenotype. A direct phenotype is defined as the observable characteristic obtained as a direct result of carrying out the SOP and retrieving a value for the parameter; using the hot plate SOP parameter of measuring the time before a first response as an example, the increased time (in seconds) for a mutant line to show a reaction compared to the baseline is a direct phenotype. An inferred phenotype is the extrapolation of the direct phenotype to infer some biological meaning. For instance, using the hotplate SOP example again, it could be inferred that the mutant line demonstrates an "increased thermal nociceptive threshold" (MP:0001973). This is an inferred phenotype because the parameter measures a time in seconds and not a behavioural threshold. Although in this case the connection between direct and inferred phenotype is relatively intuitive, in other cases it may not be and may depend on a number of phenotypic characters, represented by individual parameters, perhaps from different SOPs, before a complete phenotype can be inferred. Additionally, there may be disagreement within the community as to what biological meaning can be inferred from the results of certain parameters. For these reasons we make the distinction between direct and inferred phenotypes, where inferred phenotypes are currently defined using community recognised MP concepts.

### Data querying

Discussions with scientists during this practical ontology annotation process have revealed that there is a preference for interacting with the database, at the point of data querying, via community standard compound phenotype ontology terms where complex qualitative phenotypes are concerned. It is recognised that for some compound terms, when deconstructed into EQ format, they may lose their biological meaning. For example the term "belly spot" (MP:0000373) is deconstructed to "spotted" (PATO:0000333) *has_quality *"white" (PATO:0000323) *inheres_in *"coat hair" (MA:0000155) *part_of *"abdomen" (MA:0000029) (available from the *mammalian_phenotype logical definitions *OBO file, OBO Foundry). A solution, as implemented within EuroPhenome, is to store the phenotype in the database in the deconstructed format but allow entry of the data and subsequent querying via the compound term, so in this example *belly spot*.

## Discussion

As the use of bio-ontologies to define mouse phenotype observations becomes increasingly commonplace it is essential that the ontologies are accessible and understandable by those scientists who will make use of them and benefit from their implementation the most. This demographic is no longer restricted to ontologists or bioinformaticians, who will continue to play an essential role in developing and maintaining ontologies, but includes the "wet-science" researchers who will want to query large data sets using meaningful ontological terms and relationships in order to find phenotypes of interest. A specific example taken from the EUMODIC project would be scientists from secondary phenotyping clinics (centres that carry out more sophisticated phenotyping on lines identified as phenodeviants from first line screens) who will want to identify individual mice exhibiting relevant mutant phenotypes from EuroPhenome which will then undergo secondary phenotyping procedures. These researchers will also become increasingly responsible for entering their data into databases, albeit with appropriate quality control mechanisms in place, so the descriptive power of ontologies must be exploited to ensure they are as scientist friendly as possible.

In our discussions with phenotyping scientists with regards to interpreting their free-text descriptions of mouse phenotypes, we have identified a number of omissions of terms from the Mouse Anatomy ontology, for example *nose skin*, which were regarded as essential for the precise categorisation of phenotypes. In other cases existing terms were not intuitive to scientists and synonyms were suggested, for example "hind paw" as a synonym of "foot" (MA:0000044) and "skull" as a synonym of "head bone" (MA:0000576). Terms were also identified which required definitions in order to convey any useful meaning, for example "foot digit 1" (MA:0000465) and "hand digit 4" (MA:0000457). Our suggestions were passed onto MA curators. It is only through the practical application of phenotype ontologies that omissions and potential improvements such as these will be identified.

**Figure 3:**
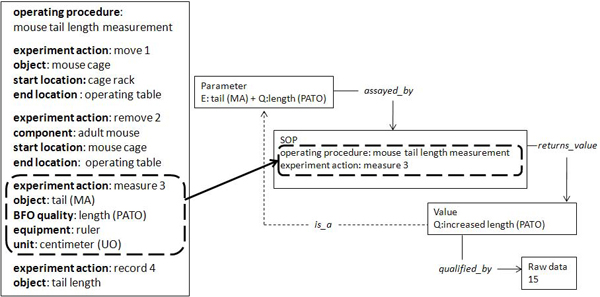
**Incorporation of assay data into the annotation framework for an individual mouse**. A simplified operating procedure is shown marked up using EXACT ontology concepts (left box). The phenotype and procedural data capture framework, to describe an instance of tail length, incorporates the experimental action from within the SOP where the phenotype data was obtained. Also shown is the relationship of parameter *assayed_by *SOP *returns_value *value, which is a child quality of the parameter quality *qualified_by *raw data.

A desirable advancement of the current ontology annotation framework would include a description of how the data for a particular parameter was derived experimentally. In order to facilitate this aim, current research is focused on the development and implementation of an assay ontology which will provide coherent definitions of each individual procedural component contained within a SOP. Work to investigate the benefits associated with representing the EMPReSS Slim SOPs using the Ontology of Experimental Actions (EXACT) [20] has begun. EXACT has been used to define each individual experimental action within a SOP (Figure 3). The context of a specific phenotypic EQ annotation can then be defined with the inclusion of this procedural data into a phenotype data capture framework as illustrated in Figure 3.

## Conclusion

With the advent of large scale mouse phenotyping programs which aim to phenotype a mouse knockout for every gene in the mouse genome, automated annotation with ontologies using a pipeline like the one described here will be crucial. In comparison to the genome sequencing projects, automated phenotype annotation can be curated by experts but an automated method of identifying the putative phenotype profile of a mouse mutant quickly is vital to allow users a window into a large dataset. We have described the ongoing efforts within the EuroPhenome mouse phenotyping resource to implement both the MP and the EQ combinatorial approach to systematically annotate real mouse phenotypes, derived from community approved SOPs, on a large scale. The four levels of annotations sees the marrying together of the two different phenotype annotation approaches into a framework that facilitates both data accessibility to mouse scientists using familiar terminology and cross-database and cross-species phenotype statement comparisons through the storage of phenotypes in the EQ format at the database level. The computational annotation of mouse mutant lines with phenotype ontologies is key to analysing large databases of raw phenotyping data. The explosion of this raw phenotyping data will mean that expert annotation of each mutant will be unfeasible. The automated approach allows the annotation to be refined as new raw data is incorporated into the database. Future interfaces for the querying of EuroPhenome data will exploit mappings between MP and EQ terms to accommodate the direct retrieval of EQ annotations in addition to querying via MP.

## List of abbreviations used

ChEBI: Chemical Entities of Biological Interest ontology

DAG: Directed Acyclic Graph

EMPReSS: European Mouse Phenotyping Resource of Standardised Screens

ENU: N-ethyl-N-nitrosourea

EQ: Entity + Quality

EUCOMM: European Conditional Mouse Mutagenesis Program

EUMODIC: European Mouse Disease Clinic

EXACT: Experiment Actions ontology

GO: Gene Ontology

KOMP: Knock Out Mouse Project

LIMS: Laboratory Information Management System

MA: Mouse Anatomy ontology

MIMPP: Minimal Information for Mouse Phenotyping Procedures

MP: Mammalian Phenotype ontology

NorCOMM: North American Conditional Mouse Mutagenesis project

OBO: Open Biomedical Ontologies

PATO: Phenotypic Quality Ontology

SOP: Standard Operating Procedure

XML: Extensible Markup Language

## Competing interests

The authors declare that they have no competing interests.

## Authors' contributions

T.B. carried out the ontology research and implementation. A-M.M. contributed to the SOP and parameter annotations. All authors read and approved the final manuscript.
